# Toxicity Effects of Perfluorooctanoic Acid (PFOA) and Perfluorooctane Sulfonate (PFOS) on Two Green Microalgae Species

**DOI:** 10.3390/ijms24032446

**Published:** 2023-01-26

**Authors:** Amin Mojiri, Mansoureh Nazari Vishkaei, Hanieh Khoshnevis Ansari, Mohammadtaghi Vakili, Hossein Farraji, Norhafezah Kasmuri

**Affiliations:** 1 Department of Civil and Environmental Engineering, Graduate School of Advanced Science and Engineering, Hiroshima University, 1-4-1 Kagamiyama, Higashihiroshima 739-8527, Japan; 2 School of Pharmacy, University of 17 August 1945, Jakarta 14350, Indonesia; 3 Department of Civil and Environmental Engineering, University of California, Davis, CA 95616, USA; 4 ORLEN UniCRE a.s., Revoluční 1521/84, 400 01 Ústí nad Labem, Czech Republic; 5 School of Physical and Chemical Sciences, University of Canterbury, Christchurch 8140, New Zealand; 6 School of Civil Engineering, College of Engineering, University Technology MARA, Shah Alam 40450, Selangor, Malaysia

**Keywords:** microalgae, toxicity, PFOA, PFOS, water

## Abstract

Amongst per- and polyfluoroalkyl substances (PFAS) compounds, perfluorooctanoic acid (PFOA) and perfluorooctane sulfonate (PFOS) have a high persistence in physicochemical and biological degradation; therefore, the accumulation of PFOS and PFOA can negatively affect aquatic organisms and human health. In this study, two microalgae species (*Chlorella vulgaris* and *Scenedesmus obliquus*) were exposed to different concentrations of a PFOS and PFOA mixture (0 to 10 mg L^−1^). With increases in the contact time (days) and the PFAS concentration (mg L^−1^) from 1 to 7, and 0.5 to 10, respectively, the cell viability, total chlorophyll content, and protein content decreased, and the decrease in these parameters was significantly greater in *Scenedesmus obliquus*. As another step in the study, the response surface methodology (RSM) was used to optimize the toxicity effects of PFAS on microalgae in a logical way, as demonstrated by the high *R^2^* (>0.9). In another stage, a molecular docking study was performed to monitor the interaction of PFOS and PFOA with the microalgae, considering hydrolysis and the enzymes involved in oxidation-reduction reactions using individual enzymes. The analysis was conducted on carboxypeptidase in *Chlorella vulgaris* and on c-terminal processing protease and oxidized cytochrome c6 in *Scenedesmus obliquus*. For the enzyme activity, the affinity and dimensions of ligands-binding sites and ligand-binding energy were estimated in each case.

## 1. Introduction

Per- and polyfluoroalkyl substances (PFAS) are a group of anthropogenic compounds that are of rising concern worldwide because of their significant impacts on the environment and on human health [1]. PFAS have the unique properties of a high surface activity and thermal stability, as well as simultaneous hydrophobicity and oleophobicity; therefore, they have been widely used in industry (in items such as textiles, furniture, kitchenware, and aqueous film-forming foam) [2]. The extreme stability of their fluoro-carbon (C-F) bond causes PFAS to be persistent against physicochemical, biological, and thermal degradation; thus, PFAS finally accumulate in aquatic environments [3]. PFAS can be categorized into two main types on the basis of their structure, consisting of a long-chain length with 8 to 12 carbon atoms and short-chain length with 4 to 7 carbon atoms [4]. Compared with the short-chain PFAS, the long-chain PFAS are more persistent in degrading and have a longer half-life.

Among the long-chain PFAS, perfluorooctane sulfonic acid (PFOS) and perfluorooctanoic acid (PFOA) have become a global concern. Up to several tens of μg L^−1^ of PFOS and PFOA have been detected in groundwater samples in several U.S. states [5]. Similarly, Pauletto et al. [6] reported up to 0.4 mg L^−1^ of PFAS in water samples in Stockholm. Several studies reported that PFOA and PFOS were the major compounds of PFAS in aquatic environments. Apart from that, Bernardini et al. [7] stated that PFOA and PFOS had been detected in surface and groundwater worldwide, as well as in the tissues of birds, marine mammals, and fish. Moreover, because of their extraordinary persistence, toxicological effects, and bioaccumulation tendencies, PFOA and PFOS can be considered intrinsic threats to organisms and human health [7].

In the past five years, studies on PFAS have mostly focused on their detection in various environments and on assessing their sources and emissions [8]. There is currently a serious concern about the effects of PFAS on living organisms, especially in aquatic environments. Hu et al. stated that there is notably less information about the toxicity impacts of PFAS on the aquatic environment, although some studies have been reported in recent years [9]. Among the aquatic microorganisms, microalgae can be applied in the bioassays of aquatic ecosystems since they are the basis of the aquatic food chain [10]. Consequently, it is important to assess the ecotoxicological effects of PFAS on microalgae in order to predicting their overall impact on the environment [11]. According to Niu et al. [12], PFAS contamination can affect microalgae and have significant effects on higher trophic levels; consequently, the effects of PFAS on microalgae should be carefully studied. In another study, Xue et al. monitored the toxicity effects of PFOA on microalgae, and the results showed that a high concentration of PFOA had negative effects on microalgae growth. In addition, the effect of PFOA on the aquatic environment and organisms was investigated by González-Naranjo et al. [13]. As shown by these previous studies, most of the toxicity investigations have been performed using a compound of PFAS and a microalgal; thus, the impacts of different PFAS compounds mixtures on different microalgae species have not been widely investigated.

Among the green microalgae in aquatic ecosystems, *Chlorella* sp. and *Scenedesmus* sp. [14] are the unicellular species. They provide a useful biological model for studying ecotoxicology owing to their high sensitivity to toxins [15]. *C. vulgaris* is a species of eukaryotic microalgae found in many natural and artificial freshwater and soil environments. It has a small cell size, a fast growth rate, and a short reproduction time [16]. In addition, *Scenedesmus obliquus* is a rapidly growing organism that may be easily cultivated in a variety of aquatic environments and wastewater effluents [17]. Therefore, *Chlorella* sp. and *Scenedesmus* sp. were selected to be examined in this study.

In terms of the biological assessments, gaining a comprehensive understanding of the interactions between the contaminants and the microalgae is a critical part of the study, and it can be accomplished using a molecular docking analysis [18]. Molecular docking simulation approaches can be applied to predict the interactions of enzymes with ligands [19]. In previous studies of the effects of PFAS on microalgae, docking simulations of the interactions between algae enzymes and PFAS have not been widely reported.

Thus, this study aimed to (1) monitor the ecotoxicology impact of PFOA and PFOS on two microalgae, and using RSM to optimize the toxicity impacts, and (2) monitor the catalytic enzyme degradation using a molecular docking simulation.

## 2. Results and Discussion

PFOS and PFOA are persistent contaminants that have negative effects on aquatic organisms. The combined impact of PFAS (PFOS and PFOA mixture) on microalgae has not been reported widely in previous studies. Thus, in the first part of this study, water was contaminated with PFOA and PFOS, resulting in total PFAS concentrations of 0.0–10.0 mg L^−1^. In the second part of the study, the interaction between the microalgae and PFOS and PFOA was simulated with the docking technique.

### 2.1. Effects of PFOA and PFOS on Microalgae

Commonly, the toxicity impact of a toxin on the cells of microalgae is assessed by the changes in the content of protein and chlorophyll and in the cell viability [20]. The dynamics of an algal population are defined by certain factors, including algal cell viability, when studying the recovery of an algal population exposed to a toxin. The recovery of the population is quicker when the impact on the algal cells is algistatic (causing a decrease in the growth rate) and not algicidal [21].

**Figure 1:**
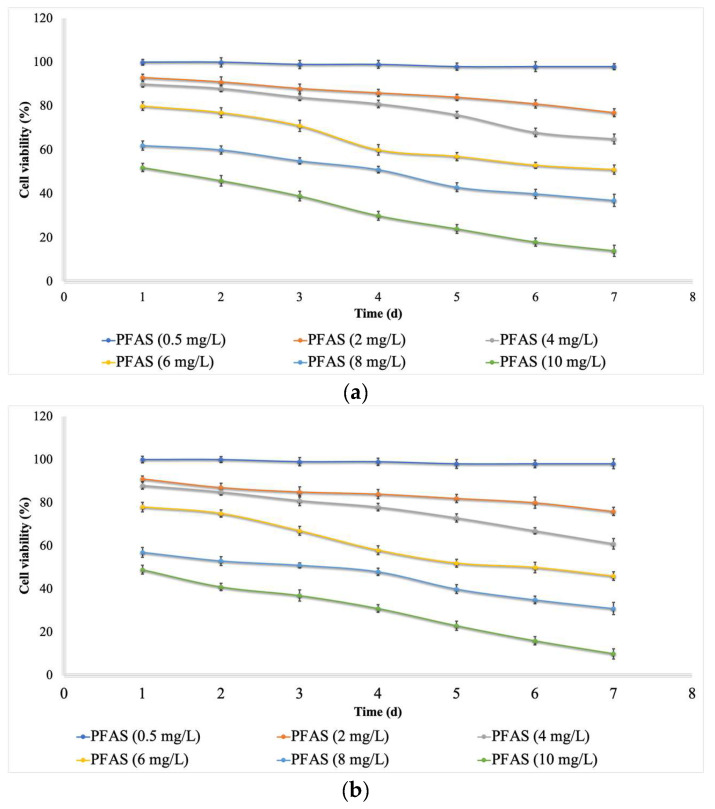
Cell viability (%) in different concentrations of PFAS (mean ± error, *n* = 3); (**a**) for *Chlorella vulgaris* and (**b**) for *Scenedesmus obliquus*.

Low concentrations (mg L^−1^) of PFAS, from 0.0 to 0.5, did not have any significant effects on the cell viability (cytotoxicity, Figure 1) of either microalgae species, while the cell viability was decreased with an increase in the concentration (mg L^−1^) from 1.0 to 10.0. Moreover, increasing the exposure time (contact time, d) caused the cell viability to decrease. For *Chlorella vulgaris*, the minimum cell viability (16%) was recorded at a concentration of 10 mg L^−1^ and a contact time of 7 days. For *Scenedesmus obliquus*, the minimum cell viability (11%) was recorded at a concentration of 10 mg L^−1^ and a contact time of 7 days. This means that a high concentration of PFAS and a long exposure time are completely toxic for microalgae growth.

Marchetto et al. [22] stated that PFOS and PFOA did not inhibit microalgae growth at a low concentration of < 1 mg L^−1^. Hu et al. [9] monitored the ecotoxicological effects of PFOA on freshwater microalgae (*Scenedesmus obliquus*). The results indicated that growth inhibition occurred with an increase in the concentration of PFOA within mg L^−1^. Liu et al. [23] reported the inhibitory effects of PFAS on the growth of algae at a PFAS concentration of up to 20 mg L^−1^. A reduction in cell viability was reported in different studies following the exposure of microalgae to organic contaminants (i.e., benzophenones, atrazine, and bisphenols) [24].

As indicated by Figure 1, the PFOA and PFOS had a more negative effect on the cell viability of *Scenedesmus obliquus* compared with that of *Chlorella vulgaris*. El-Sheekh et al. [25] reported a higher growth rate for *Chlorella vulgaris* in comparison with *Scenedesmus obliquus* after exposure to high concentrations of organic contaminants, which agrees with the current study.

Chlorophyll plays a vital role in microalgal photosynthesis, capturing light and conveying electrons [26]. In addition, proteins are key products of photosynthesis in plants and algae. Any change in the growth of microalgae through exposure to toxins can be determined by measuring its impact on chlorophyll biosynthesis and proteins [27,28].

**Figure 2:**
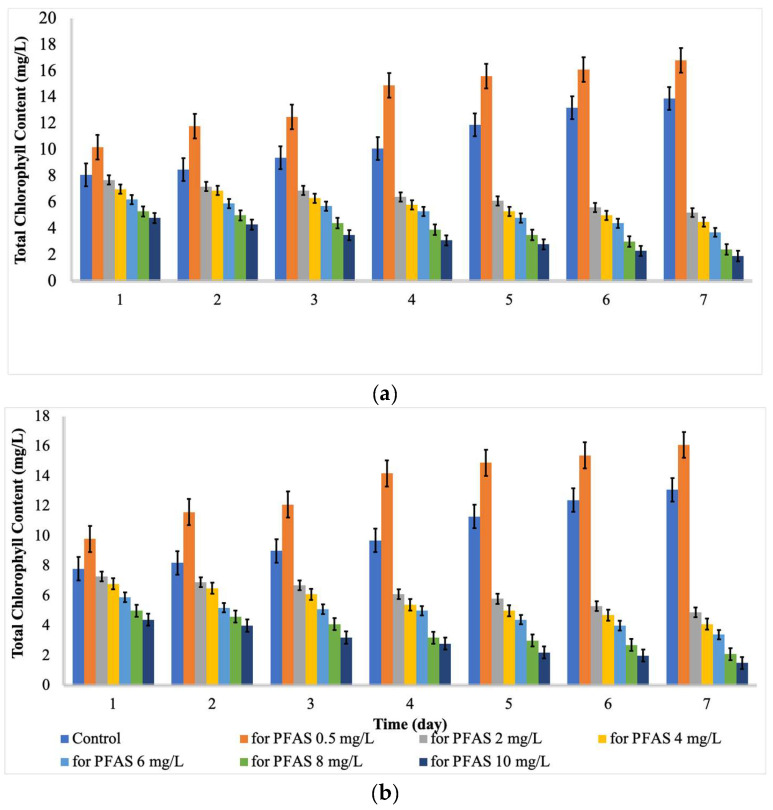
Total Chlorophyll (mg L^−1^) at different concentrations of PFAS (mean ± error, *n* = 3); (**a**) for *Chlorella vulgaris* and (**b**) for *Scenedesmus obliquus*.

**Figure 3:**
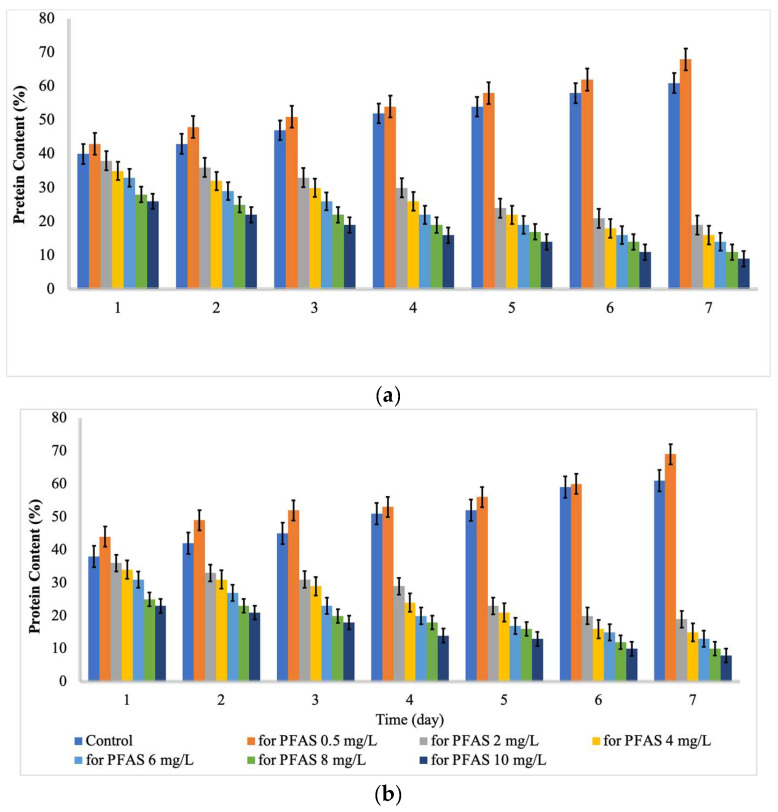
Protein content (%) at different concentrations of PFAS (mean ± error, *n* = 3); (**a**) for *Chlorella vulgaris* and (**b**) for *Scenedesmus obliquus*.

As indicated by Figure 2 and Figure 3, the content of protein and chlorophyll increased with increases in the PFAS concentration from 0 to 0.5 mg/L. Then, the content of protein and chlorophyll decreased with further increases in the contact time and PFAS concentration. For *Chlorella vulgaris*, the maximum total chlorophyll (16.8 mg L^−1^) and protein content (68%) were recorded at a PFAS concentration (mg L^−1^) of 0.5 and a contact time (d) of 7. In contrast, the minimum total chlorophyll (1.9 mg L^−1^) and protein content (9 %) were recorded at a PFAS concentration (mg L^−1^) of 10.0 and a contact time (d) of 7. For *Scenedesmus obliquus*, the maximum total chlorophyll (16.1 mg L^−1^) and protein content (69%) were reached at a PFAS concentration (mg L^−1^) of 0.5 and a contact time (d) of 7, while the minimum total chlorophyll (1.5 mg L^−1^) and protein content (8 %) were detected at a PFAS concentration (mg L^−1^) of 10.0 and a contact time (d) of 7.

Hu et al. [29] stated that chlorophyll could be considered as an indicator of growth responses to PFAS concentrations and durations. In a study, the content of chlorophyll *a* in microalgae exposed to PFOA decreased with increases in the contact time [29]. Besides that, Liu et al. [23] stated that the total chlorophyll of algae exposed to perfluorobutane sulfonic acid (PFBS) was considerably lower than that of the control on the 4th day and the 12th day, which is in line with the current findings. Hu et al. [29] reported that some protein levels were decreased after 6 d of exposure to 1–50 mg L^−1^ of PFOA. Mojiri et al. [30] reported that emerging contaminants with low concentrations could increase the protein and chlorophyll content because of an increase in the enzyme synthesis or other energy-producing fractions, while the high concentration of organic contaminants can reduce the chlorophyll and protein content due to abiotic stress damages. Upon exposure to contaminants, reactive oxygen species (ROS) cause the synthesis of chlorophyll and proteins to be blocked, ultimately damaging the structures of algal cell and even resulting the death of cells [28].

In subsequent step, the effects of PFAS on two microalgae species were optimized using RSM. RSM is a method of mathematical and statistical analysis that reveals how the total chlorophyll, protein content, and cell viability (responses) are affected by the independent factors (variables, including exposure time (A) and concentration of PFAS (B)). The statistical results for the present study are shown in Table 1 and Table 2, representing *Chlorella vulgaris* and *Scenedesmus obliquus*, respectively. As shown in Table 1 and Table 2, the high values of *R^2^* (> 0.9) indicate that RSM can optimize the effects of PFAS on microalgae in a rational way. Apart from that, the final equations (in the actual code) for the cell viability, protein content, and total chlorophyll are displayed as Equations (1)–(3) for *Chlorella vulgaris* and as Equations (4)–(6) for *Scenedesmus obliquus*.

Effects on Cell viability = 104.30 − 1.36A − 2.46B − 0.55AB + 0.02A^2^ − 0.20B^2^ (1)

Effects on Protein content = 11.62 + 0.58A − 2.05B − 0.11AB − 0.02A^2^ + 0.15B^2^ (2)

Effects on Chlorophyll = 53.06 − 0.13A − 7.42B − 0.43AB + 0.05A^2^ + 0.57B^2^ (3)

Effects on Cell viability = 103.60 − 0.61A − 3.76B − 0.56AB − 0.06A^2^ − 0.10B^2^ (4)

Effects on Protein content = 53.67 − 0.50A − 8.08B − 0.40AB + 0.09A^2^ + 0.61B^2^ (5)

Effects on Chlorophyll = 11.36 + 0.49A − 2.03B − 0.11AB − 0.01A^2^ + 0.15B^2^ (6)

Based on the RSM simulation, the maximum total chlorophyll (16.0 mg L^−1^), protein content (61.5 %), and cell viability (96 %) can be reached at a contact time of 6.9 d and a PFAS concentration of 0.8 mg L^−1^ for *C. vulgaris*, while the maximum total chlorophyll (15.7 mg L^−1^), protein content (60.9 %), and cell viability (95 %) can be reached at a contact time of 6.8 d and a PFAS concentration of 0.8 mg L^−1^ for *S. obliquus*.

### 2.2. Molecular Docking

**Figure 4:**
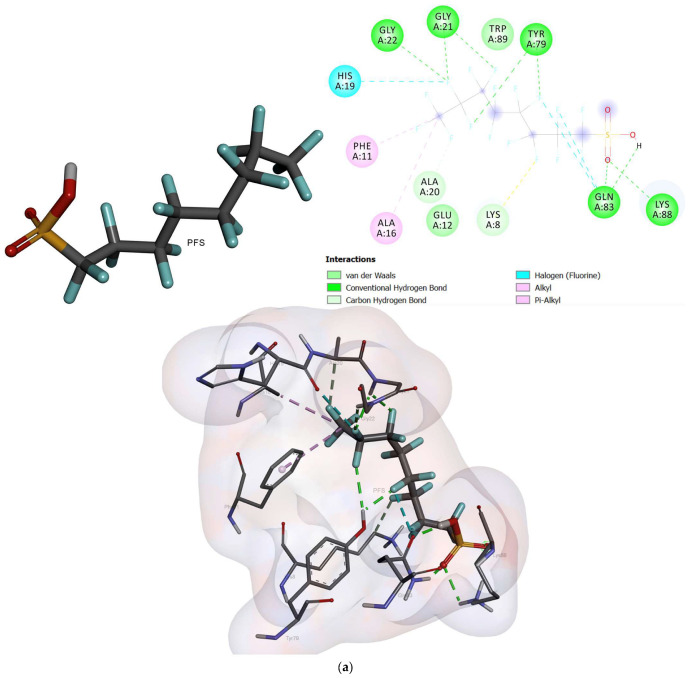

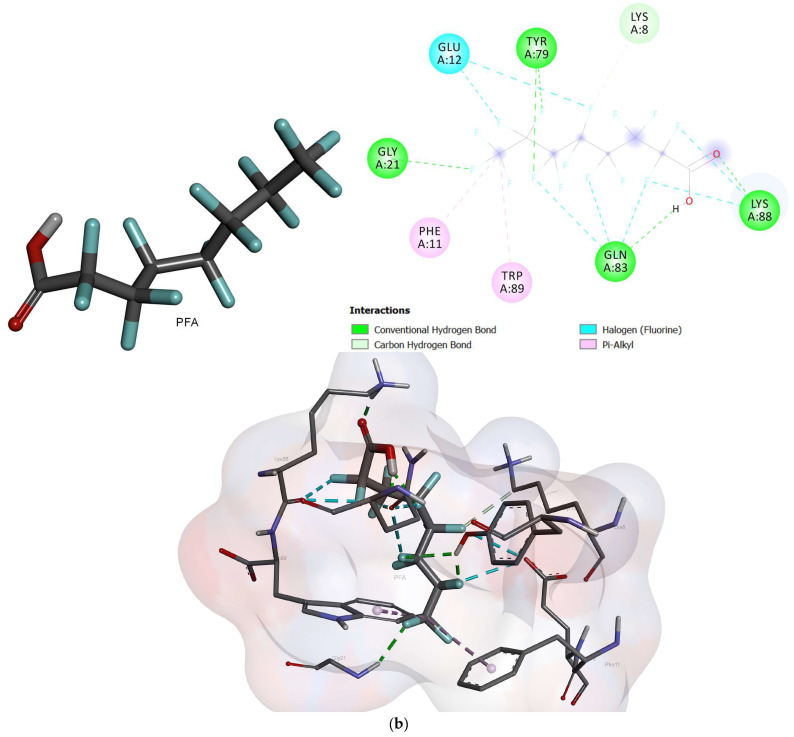
The interactions between oxidized cytochrome c6 and ligands; (**a**) PFOS and (**b**) PFOA.

**Figure 5:**
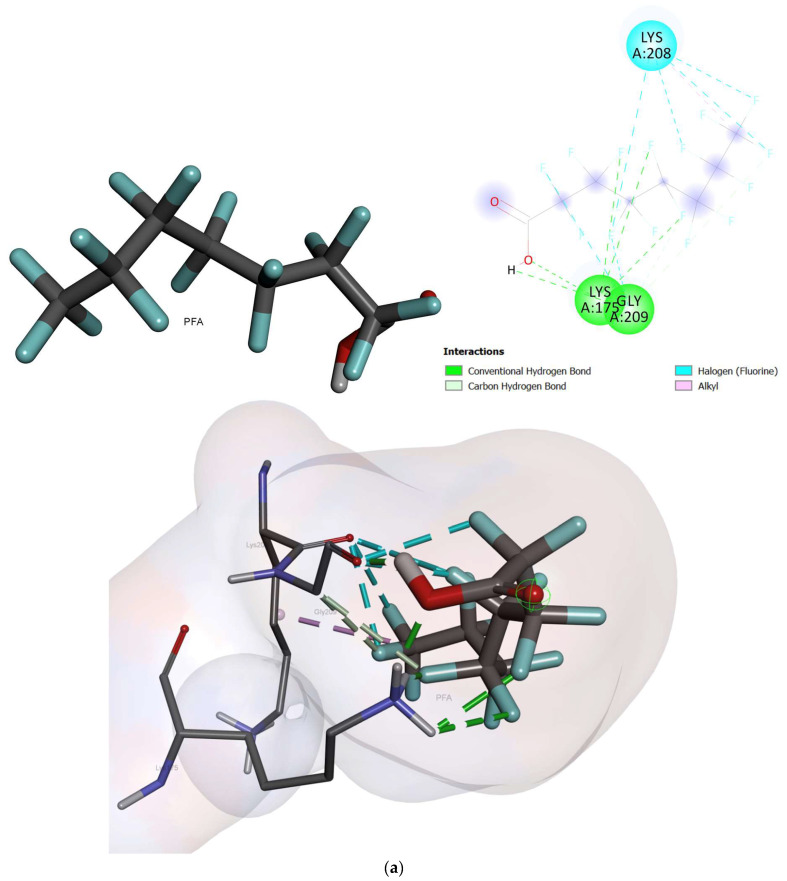

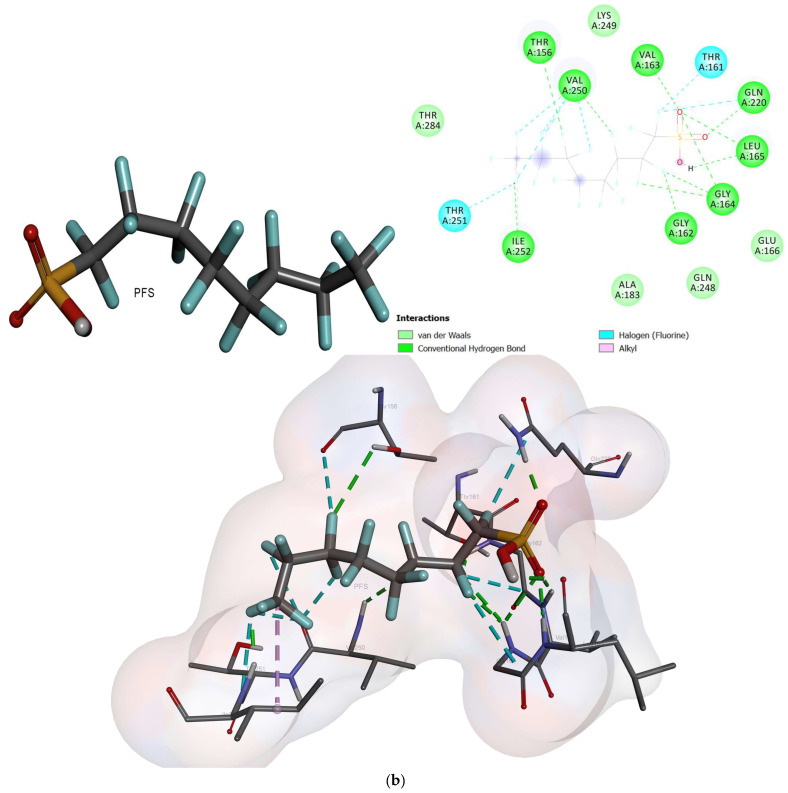
The interactions between photosystem ii d1 c-terminal processing protease and ligands; (**a**) PFOS, and (**b**) PFOA.

**Figure 6:**
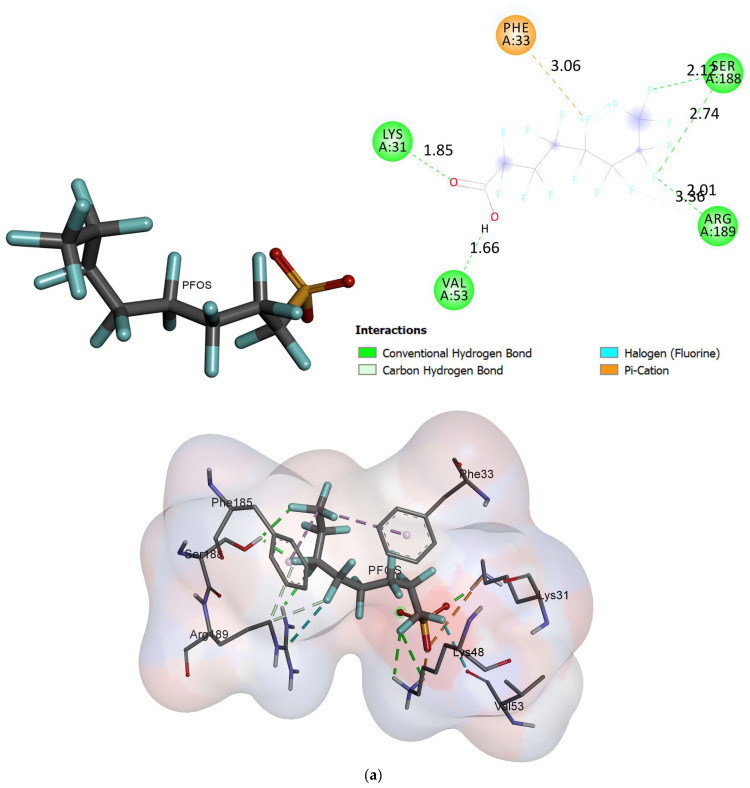

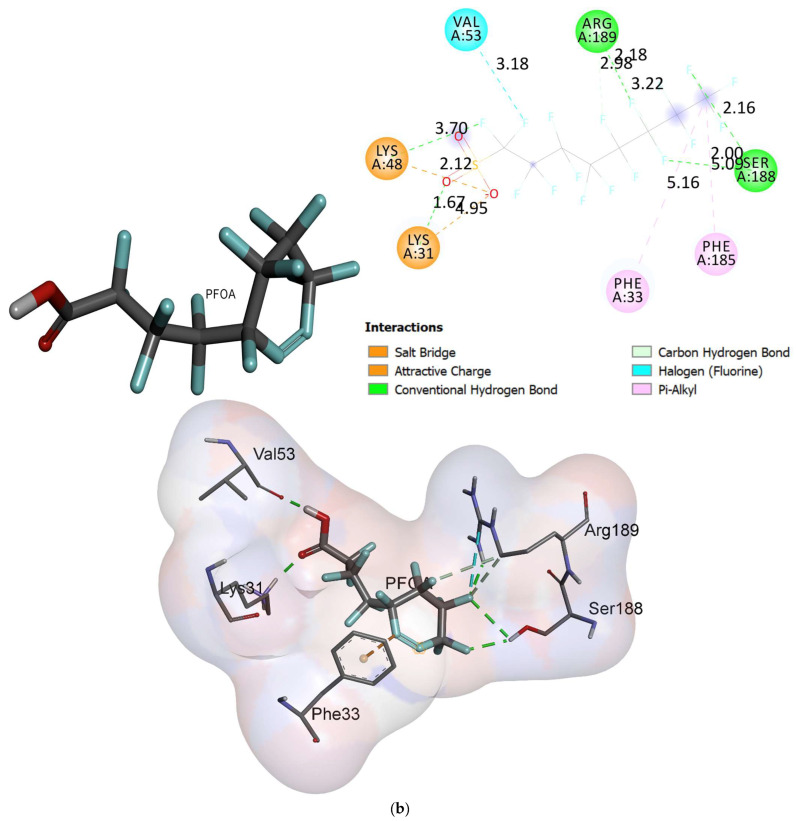
The interactions between carboxypeptidase t and n-sulfamoyl-l-lysin in vulgaris and ligands; (**a**) PFOS, and (**b**) PFOA.

The docked conformation of oxidized cytochrome c6 and photosystem ii d1 c-terminal processing protease with an active conformation of each ligand demonstrated several interactions (Figure 4, Figure 5 and Figure 6). The results for the free binding energy of each compound after interactions with different proteins are demonstrated in Table 3. 

Cytochrome 6, after interaction with PFOA, showed four hydrogen bonds with LYS8, GLN83, TYR79, and GLY21. It showed a halogen bond with GLU12 and two alkyl bonds with TRP89 and PHE11. In addition, it demonstrated carbon-hydrogen bonds with LYS8 and ALA20 with a free binding energy of -1.98 Kcal/mol [31]. The PFOS compound, after interaction with cytochrome 6, showed five hydrogen bonds with GLY22, GLY21, TYR79, GLN83, and LYS88. Also, it displayed a carbon-hydrogen bond with ALA20 and a halogen bond with HIS19. Moreover, two alkyl bonds were formed with PHE11 and ALA16 with a free binding energy of −2.42 Kcal/mol. *C. vulgaris*, after interaction with PFOA, indicated two hydrogen bonds with SER188 and ARG189.

The chemical interaction between photosystem ii d1 c-terminal processing protease and the studied compounds is demonstrated in Figure 5. The chemical interactions between the ligands and protease proteins are demonstrated in Figure 5. The PFOA compound showed two hydrogen bonds with GLY209 and LYS175. Furthermore, it displayed a halogen bond with LYS208 with a free binding energy of −2.83 Kcal/mol. The PFOS compound, after interaction with protease, revealed eight hydrogen bonds with VAL250, GLY164, LEU165, VAL163, GLN220, GLY162, THR156, and ILE252. In addition, it showed two halogen bonds with THR161 and THR251. Moreover, it revealed an alkyl bond with ILE252 with a free binding energy of -3.22 Kcal/mol [32].

*C. vulgaris*, after interaction with PFOA, demonstrated two hydrogen bonds with SER188 and ARG189. After interacting with PFOS, it showed two hydrogen bonds with SER188 and ARG189. A salt bridge was formed via LYS48 and LYS31 and the sulfonate group in PFOS (Figure 6).

In this study, the enzymes in the molecular docking analysis were selected due to their presence in the studied green algae. Molecular docking studies using the selected proteins in *Scenedesmus obliquus* suggested that these proteins can interact with PFOA and PFOS with negative free binding energy [33]. As shown in Figure 6, the interaction of carboxypeptidase t with n-sulfamoyl-l-lysin in *vulgaris*, as the another studied microalgae, after exposure to PFOA or PFOS, can complete the interaction of *Scenedesmus obliquus* with different fluorines (Table 3).

## 3. Materials and Methods

PFOA and PFOS with a purity of >95% (Table 4) were purchased from the Sigma-Aldrich Co. (Petaling Jaya, Malaysia) *Chlorella vulgaris* and *Scenedesmus obliquus* were collected from a photobioreactor in our laboratory. The stock solutions of PFOA and PFOS were prepared individually in methanol as described by Fan et al. [34]. Therefore, the low concentration of methanol in the blank (control) experiments was considered as well.

### 3.1. Experiments for the Toxicity Effects of PFAS on Microalgae

The Erlenmeyer flasks (with the amount of 5 × 10^3^ cells/mL for each microalgae) [9] were exposed to varying concentrations of the PFOA and PFOS mixture (0.0 mg L^−1^ to 10.0 mg L^−1^) at varying exposure times (0 to 7 d). *Chlorella vulgaris* and *Scenedesmus obliquus* were cultivated in an Erlenmeyer flask containing BG11 medium. Dark:light cycles of 8:16 with an illumination intensity of 90 ± 20 μmol photons m^−2^ s^−1^ were considered optimal for algae culture at a temperature of 25 ± 2 °C and neutral pH.

### 3.2. Cytotoxicity Rate, and Content of Protein and Chlorophyll

Cytotoxicity (%, Equation (7)) was assessed as described by Namasivayam et al. [35] based on the inhibition of cell growth. Firstly, different concentrations (mg L^−1^) of the PFOS and PFOA mixtures (0.0 to 10) were considered to determine an algal mortality of 50%, and then the daily cell growth was tracked for up to 5 days in all samples. The selected concentrations were in line with previous studies [23]. Cell growth was inhibited by nearly 50% at a concentration of PFAS of 8.0 mg L^−1^. Secondly, the cell growth rate was detected and monitored at a concentration of PFAS of 8.0 mg L^−1^ over 15 d and compared with the control (blank sample) through spectrophotometry (measuring optical density (OD)) at a 630 nm wavelength [10].

(7) $$\mathrm{Cytotoxicity}\;(\%)=\frac{\left(OD\;of\;individual\;test\;group\right)100}{\left(OD\;of\;control\;group\right)}$$

The content of protein and chlorophyll was tested using a UV–vis spectrophotometer (UV-1280, Shimadzu, Japan). The samples were prepared for the experiments by collecting 10 mL of culture with centrifugation at 4500 rpm for 15 min [36]. For testing chlorophyll, the samples were investigated at wavelengths 646 nm and 663. Then, the chlorophyll content (Ch) was estimated by Equations (8)–(10) [37].

Ch a (μg mL^−1^) = 12.25 (A_663_) – 2.55 (A_646_) (8)

Ch b (μg mL^−1^) = 20.13 (A_646_) − 4.91 (A_663_) (9)

Ch a + b (μg mL^−1^) = 17.76 (A_646_) + 7.34 (A_663_) (10)

where A_646_ and A_663_ display the absorbance at wavelengths of 646 nm and 663 nm, respectively.

The protein and chlorophyll content were investigated at wavelengths of 260 nm and 280 nm. Then, the protein content was estimated using Equation (11) [38].

*Protein content* = (1.55 × A_280_) − (0.77 × A_260_) (11)

where A_260_ and A_280_ display the absorbance at wavelengths of 260 nm and 280 nm, respectively.

### 3.3. Analyses the PFAS in Aqueous Solution

The high-pressure liquid chromatograph (LC-20AT, Shimadzu, Tokyo, Japan) with a CDD-10AVP conductivity detector and a C18 column was used to detect the PFAS concentrations. The mobile phase was composed of methanol and ammonium acetate [39], and the flow rate was 0.3 mL/min. Then, the standard deviation of the baseline waves in three replications was applied to measure the limit of detection (LOD) [40].

### 3.4. Statistical Analysis and Optimization Process

The optimization and statistical analysis were conducted using central composite design (CCD) and response surface methodology (RSM) through DOE 10.0.7 with two independent factors, the exposure time (day), and the initial concentration of PFAS (mg L^−1^), under the quadratic model (Equation (12)). A P-value (probability) with a 95% level of confidence was considered.

(12) $$Y=\beta_{0}+\overset{k}{\underset{j=1}{\sum }}\beta_{j}X_{j}+\overset{k}{\underset{j=1}{\sum }}\beta_{jj}X^{2}+\underset{i}{\sum }\overset{k}{\underset{<j=2}{\sum }}\beta_{ij}X_{i}X_{j}+e_{i}$$

where *Y* and *e* represent response and error, respectively. The interaction coefficients of the linear, quadratic, and second order terms are designated as *β_j_*, *β_jj_*, and *β_ij_*, respectively, and *X_i_* and *X_j_* are variables.

### 3.5. Protein Preparation for Bioinformatics Analysis

#### 3.5.1. Software

From the websites www.phyton.com (accessed on 10 December 2022), http://mgltools.scripps.edu (accessed on 10 December 2022), http://autodock.scripps.edu (accessed on 10 December 2022), http://accelrys.com (accessed on 10 December 2022), and https://acms.ucsd.edu (accessed on 10 December 2022), molecular graphics laboratory (MGL) tools, AutoDock4.2, Bio Via draw, Discovery studio visualizer 2017 and Chem3D were downloaded, respectively.

#### 3.5.2. Methods

**Figure 7:**
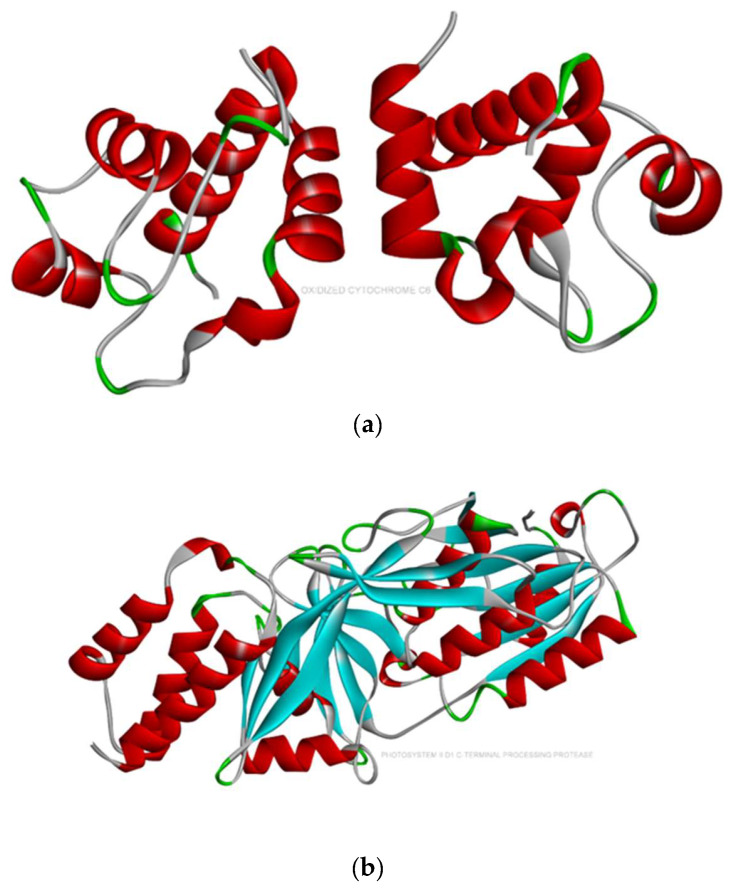

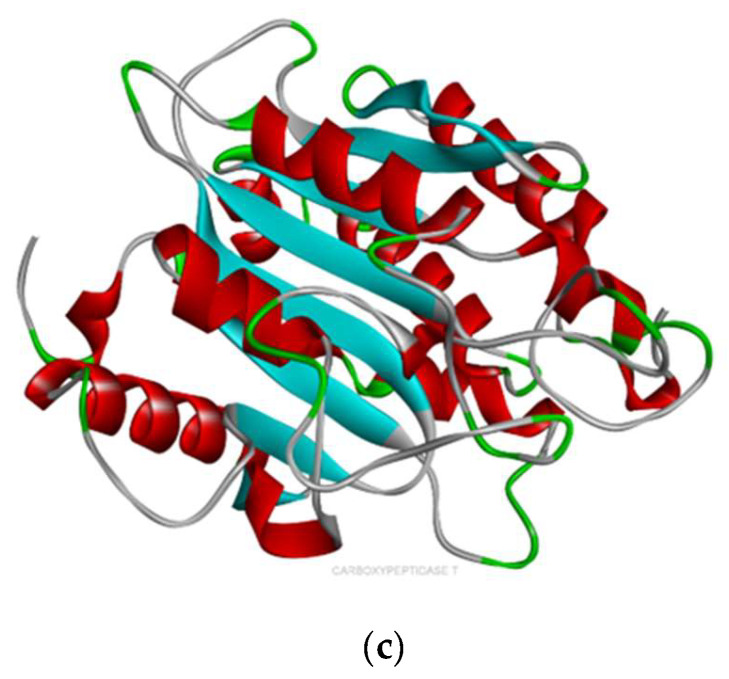
(**a**) c-terminal processing protease (PDB ID: 1FC7); (**b**) oxidized cytochrome c6 from *Scenedesmus obliquus* (PDB ID: 1C6O); and (**c**) carboxypeptidase t with n-sulfamoyl-l-lysin (PDB ID: 6T9Y) in *C. vulgaris*.

From the Protein Data Bank (www.rvcsb.org/pdb; accessed on 10 December 2022), the three-dimensional crystal structure of photosystem ii d1 c-terminal processing protease with the PDB ID: 1FC7, oxidized cytochrome c6 from *Scenedesmus obliquus* with the PDB ID: 1C6O, and carboxypeptidase in *C. vulgaris* with the PDB ID (6T9Y) were obtained (Figure 7) [41]. Using Argus Lab, all non-essential molecules of water and heteroatoms were deleted from the complexes bound to the receptor molecule, and then hydrogen atoms were augmented to the target receptor molecule.

#### 3.5.3. Ligand Preparation

Using the recognized structure of the ligands from crystallography, the synthetic active compounds of PFOA and PFOS were applied using Pubchem in the SDF format, converted to the PDB format using Pymol, and further applied for the docking studies.

Using the AutoDock tools, the preliminary structures of the proteins were organized [42]. After deleting molecules of water, polar hydrogen and Kollman charges were added to the preliminary structure of protein. The grid rid box was set to a size of 126 × 126 × 126 Å and a grid spacing of 0.375 Å at the binding site. The preliminary structure for all of the ligands, named PFOA and PFOS, was assembled using BioVia draw [43]. Using Autodock Tools, Gasteiger charges were assigned to the optimized ligands. A total of 100 docking runs were carried out, with a crossover rate of 0.8 and a mutation rate of 0.02. The population size was set to 250 randomly placed individuals. Lamarckian Genetic was employed as the searching algorithm with a translational phase of 0.2 Å, a quaternion step of 5 Å, and a torsion step of 5 Å [44].

## 4. Conclusions

The toxicity effect of PFOA and PFOS on *Chlorella vulgaris* and *Scenedesmus obliquus* was investigated. The findings of the study are summarized below:

1. The minimum cell viability (16%) and content of chlorophyll (1.9 mg L^−1^) and protein (9%) for *Chlorella vulgaris* were detected at high concentrations of the PFOS and PFOA mixture (10 mg L^−1^) and at a long exposure time (contact time, 7 days).
2. The minimum cell viability (11%) and content of chlorophyll (1.5 mg L^−1^) and protein (8%) for *Scenedesmus obliquus* were reported at high concentrations of the PFOS and PFOA mixture (10 mg L^−1^) and at a long exposure time (contact time, 7 days).
3. In comparison to *Chlorella vulgaris*, *Scenedesmus obliquus* is more sensitive to exposure to PFAS.
4. Based on the molecular docking investigation, *C. vulgaris* and *Scenedesmus obliquus* can potentially interact with PFOA and PFOS more efficiently when utilized together due to the presence of the examined enzymes. This may reduce the toxic effects of PFAS in water resources. However, further investigation is needed to confirm this hypothesis.

## Figures and Tables

**Table 1:** Statistical analysis results for response parameters for *C. vulgaris*.

Reponses	R^2^ *	Adj. R^2^	Adec. P	SD	CV
Cell viability	0.987	0.985	78.94	2.98	4.37
Protein content	0.900	0.891	29.8	3.91	8.5
Total chlorophyll	0.900	0.892	29.4	4.03	9.3

* R^2^: R-square, Adj. R^2^: Adj R-square, Adeq Precision, SD: Std. Dev.; C.V %.

**Table 2:** Statistical analysis results for response parameters for *S. obliquus*.

Reponses	R^2^ *	Adj. R^2^	Adec. P	SD	CV
Cell viability	0.985	0.983	74.93	3.23	4.92
Protein content	0.907	0.902	28.8	6.31	6.31
Total chlorophyll	0.907	0.901	29.83	1.73	8.60

* R^2^: R-square, Adj. R^2^: Adj R-square, Adeq Precision, SD: Std. Dev.; C.V %.

**Table 3:** Free binding energy and Ki of interaction between photosystem ii d1 c-terminal processing protease, oxidized cytochrome c6 proteins, and carboxypeptidase t with n-sulfamoyl-l-lysin in vulgaris and ligands.

FbE(Kcal/mol)	Vug	Cyt6	Protease
PFOA	−2.96	−1.98	−2.83
PFOS	−3.07	−2.42	−3.22
Ki	Vug	Cyt6	Protease
PFOA	6.75 mM	35.58 mM	8.42 mM
PFOS	5.60 mM	16.90 mM	4.38 mM

**Table 4:** Characteristics of PFOA and PFOS.

Compounds	Chemical Formula	Chemical Structure	Molecular Weight (g/mol)	CAS Number
PFOA	CF_3_(CF_2_)_6_COOH	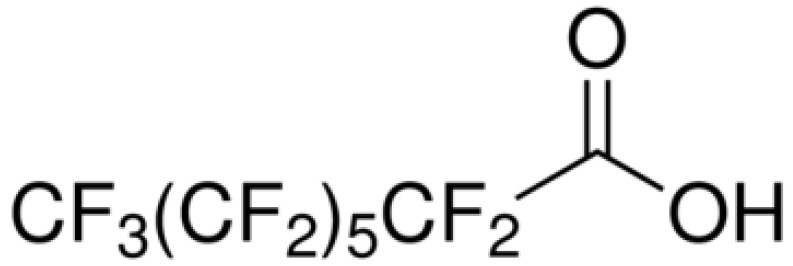	414.07	335-67-1
PFOS	CF_3_(CF_2_)_7_SO_3_H	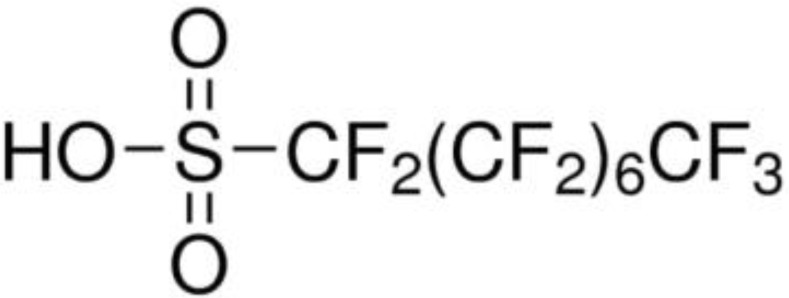	500.13	1763-23-1

## Data Availability

Not applicable.
